# Treatment of anterior shoulder instability: a bibliometric analysis

**DOI:** 10.1186/s13018-022-02913-z

**Published:** 2022-01-15

**Authors:** Mingtao Zhang, Zhitao Yang, Borong Zhang, Tao Liu, Xiangdong Yun

**Affiliations:** Department of Orthopaedics, Lanzhou University Second Hospital, No. 82 Cuiyingmen, Chengguan District, Lanzhou, 730030 Gansu China

**Keywords:** Anterior shoulder instability, Treatment, Bibliometric analysis, Reconstruction

## Abstract

**Purpose:**

The treatment of anterior shoulder instability is a focus in the field of sports medicine. While much research has been conducted, few bibliometric studies have been performed in this field. This study analyzed the main characteristics and identified emerging research trends and hotspots related to the treatment of anterior shoulder instability over the past four decades.

**Methods:**

We searched for (anterior shoulder instability OR anterior shoulder dislocation) AND (treatment OR reconstruction) in ARTICLE (Mesh) in the Web of Science database from 1980 to 2020. We analyzed the keywords, author, institution, country, number of citations, average number of citations, publication year, and partnership of the identified articles. Information about annual publications was analyzed using Microsoft Excel 2019; the remaining data were analyzed using VOSviewer version 1.6.11 (Leiden University, Leiden, Netherlands) and CiteSpace version 5.7.R2 (Drexel University, Philadelphia, PA, USA).

**Results:**

A total of 1964 articles were published between 1980 and 2020. The *American Journal of Sports Medicine*, the United States, the United States Department of Defense, and Arcieio were journals, countries, institutions, and authors with the highest numbers of publications. The topic hotspots were instability, shoulder, and dislocation, while the research frontiers were arthroscopic, Bankart repair, Latarjet procedure, risk factors, recurrence, and complications.

**Conclusion:**

The treatment of anterior shoulder instability has shown an increasing number of publications each year and achieved great progress. The United States made the most outstanding contributions to this important field. Arthroscopic, Bankart repair, and Latarjet procedures were research hotspots and risk factors, recurrence, and complications were likely to research frontiers.

## Introduction

The shoulder joint is one of the most commonly dislocated joints in the human body, with an incidence rate of approximately 1.7% [1, 2]. Since the articular surface of the glenohumeral joint faces the anterior inferior side and the anterior joint capsule is weaker, anterior shoulder dislocation is most commonly seen in the clinic. Anterior shoulder instability contributes 90% of total instability [3]. Maintaining the stability of the shoulder joint is a complex interaction between both static and dynamic factors. The static factors include the glenoid labrum, rotator interval, glenohumeral ligament, and glenohumeral articulation. In addition, the dynamic factors include the rotator cuff and scapular muscles.

In recent years, with the development of arthroscopic instruments and surgical techniques, shoulder arthroscopic surgery has become the main method for the treatment of most shoulder dislocation, and can achieve a reconstruction effect equivalent to or better than that for open surgery [4, 5].

Bibliometric analysis is a statistical method used to evaluate published literature [6]. Bibliometric methods have been developed and widely applied in natural sciences and social sciences, and can help researchers understand the publishing trends of relevant knowledge, involving research results in specific countries or regions [7]. Bibliometric analysis can illustrate information of a certain field, including journals, authors, institutions, countries, and keywords. CiteSpace was first developed by Chaomei Chen in 2004 and is the most commonly used analysis software [8]. In addition, the VOS viewer software developed by the Science and Technology Research Center of Leiden University in the Netherlands can be used for the cluster, overlay, and density views of the literature to evaluate the research direction and hotspots in the literature [9]. Therefore, the present study used CiteSpace and VOS viewer to perform a bibliometric analysis of publications on the treatment of anterior shoulder instability.

The purposes of our study were to report on scientific output in research on the treatment of shoulder instability from the Web of Science (1980–2020), analyze the main characteristics of related articles, and identify emerging research trends and hotspots.

## Methods

### Data collection and search strategy

Articles on the treatment of shoulder instability were retrieved from the Web of Science Core Collection (WoSCC) from 1980 to 2020. The search strategy was as follows: TS = (anterior shoulder instability OR anterior shoulder dislocation) AND (treatment OR reconstruction) AND LANGUAGE (English) AND DOCUMENT TYPES (Article).

**Fig. 1:**
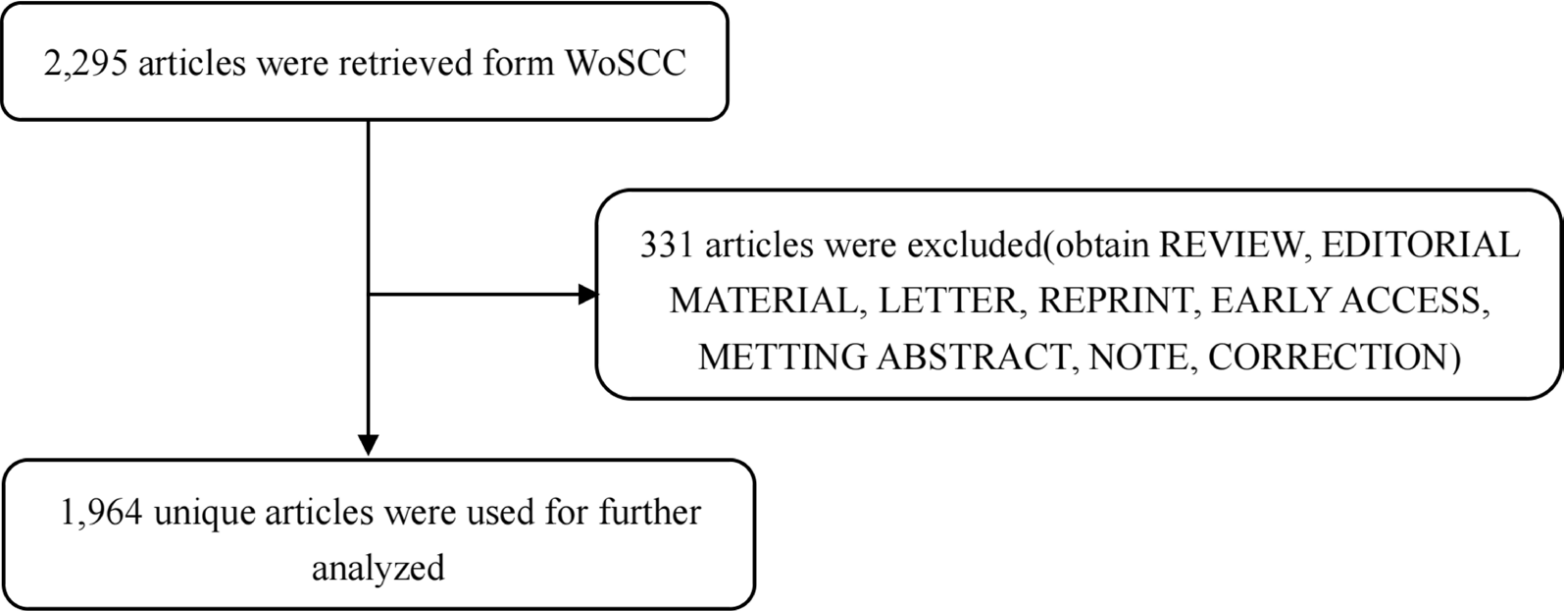
Flowchart of the treatment of anterior of shoulder instability research

Among a total of 2295 articles retrieved; those of the following types were excluded: REVIEW, EDITORIAL MATERIAL, LETTER, REPRINT, EARLY ACCESS, MEETING ABSTRACT, NOTE, CORRECTION. A total of 1,964 unique articles were included in the subsequent further analyses. After extraction, the data retrieved from WoSCC were downloaded and transferred into CiteSpace and VOS viewer software for bibliometric analysis. The retrieval strategy used in this study is shown in Fig. 1.

### Statistical analysis

Information about publications, including annual publications and journal distributions, was obtained from the WoSCC database. The corresponding chart of publications was generated using Microsoft Excel 2019, while Cite Space and VOS viewer software were used to visualize the network analysis of the literature related to the treatment for shoulder instability.

## Results

### Basic information

**Fig. 2:**
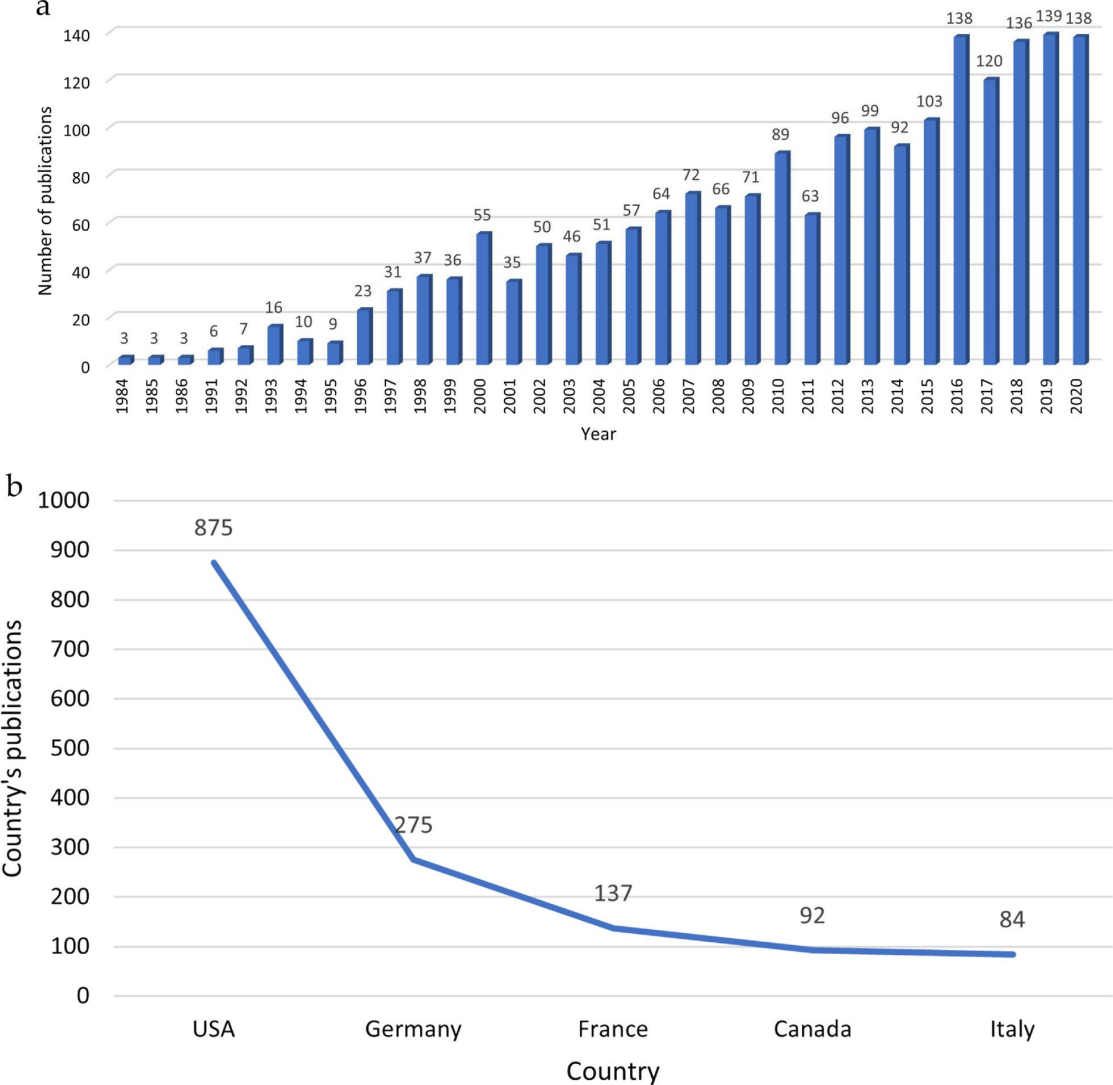
**a** The number of annual articles, and **b** Country’s publications

A total of 1964 articles were retrieved. The number of annual articles about the treatment of anterior shoulder instability increased from 3 in 1984 to 138 in 2020, with the number of annual articles peaking in 2019 (Fig. 2a). The United States had the most publications (44.5%), followed by Germany (14.0%), France (6.9%), Canada (4.7%), and Italy (4.3%) (Fig. 2b).

### Distribution of journals

The 1964 articles were published in a total of 234 journals, and the top 10 journals with the most publications are listed in Table 1. The journals in order of decreasing numbers of publications were the *American Journal of Sports Medicine*, accounting for approximately 11.4%, followed by the *Journal of Shoulder and Elbow Surgery* (11.2%) and *Arthroscopy: The Journal of Arthroscopic and Related Surgery* (9.2%). Only the *American Journal of Sports Medicine* had an impact factor (IF) above 5 (IF = 5.81).

**Table 1:** The top 10 journals in the treatment of anterior shoulder instability

Journal	Frequency	Country/region	IF*
American Journal of Sports Medicine	223	USA	5.81
Journal of Shoulder And Elbow Surgery	220	USA	2.817
Arthroscopy-The Journal of Arthroscopic And Related Surgery	181	USA	4.325
Knee Surgery Sports Traumatology Arthroscopy	100	Germany	3.166
Journal of Bone And Joint Surgery-American Volume	88	USA	4.578
Arthroscopy techniques	75	USA	1.9
Operative Techniques In Sports Medicine	56	USA	0.35
Archives of Orthopaedic And Trauma Surgery	46	Germany	2.021
Orthopaedic Journal of Sports Medicine	44	USA	2.492
Clinical Orthopaedics And Related Research	41	USA	4.329

**Fig. 3:**
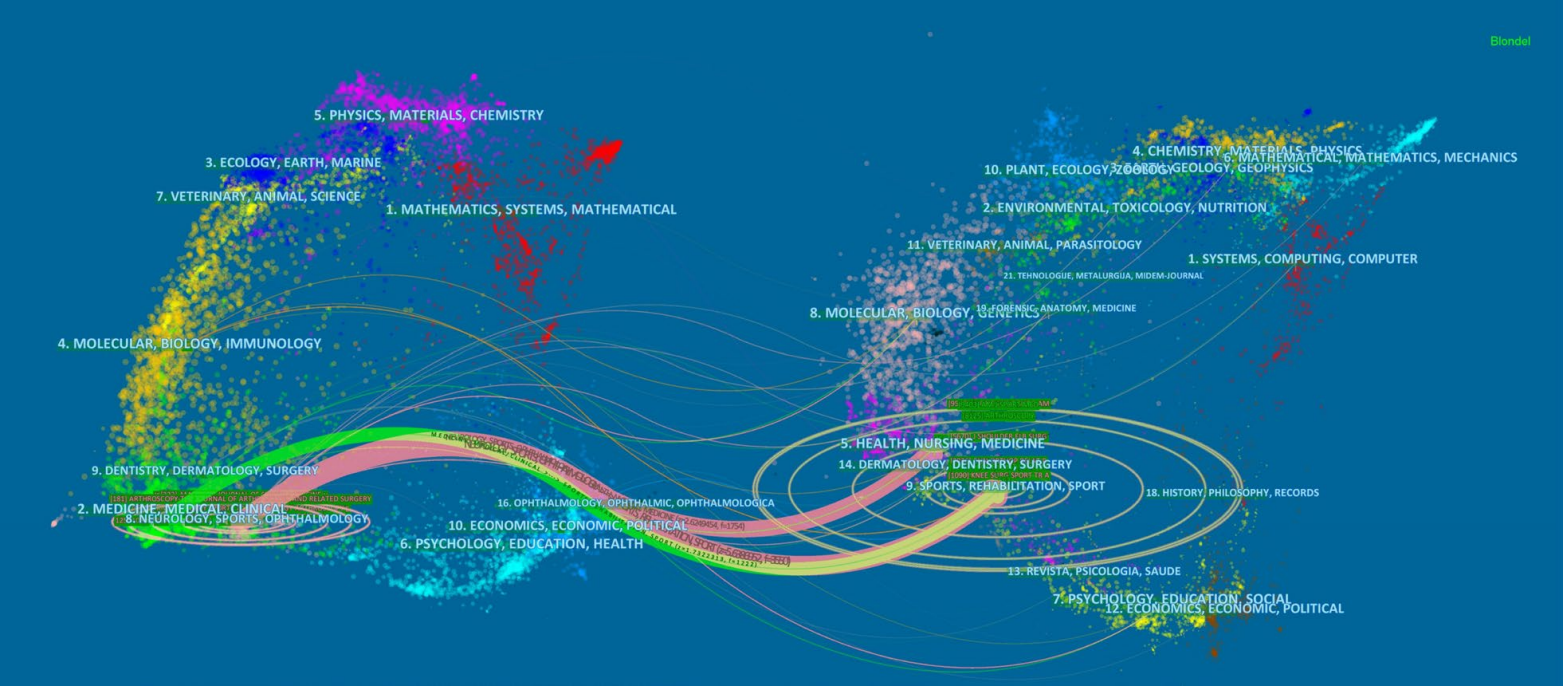
The dual-map overlay of journals related to anterior shoulder instability

The left part of the dual-map in Fig. 3 indicates the fields that have published articles on the treatment of anterior shoulder instability, while the right part indicates the fields from which the references originated. Most articles on the treatment of anterior shoulder instability were published in the fields of medicine, clinical, neurology, and sports and cited journals in the fields of sports, rehabilitation, health, nursing, and medicine. The center of the circle indicates that the greater the importance, the thicker the line, the closer cooperation between different fields.

### Distributions of countries and institutions

**Fig. 4:**
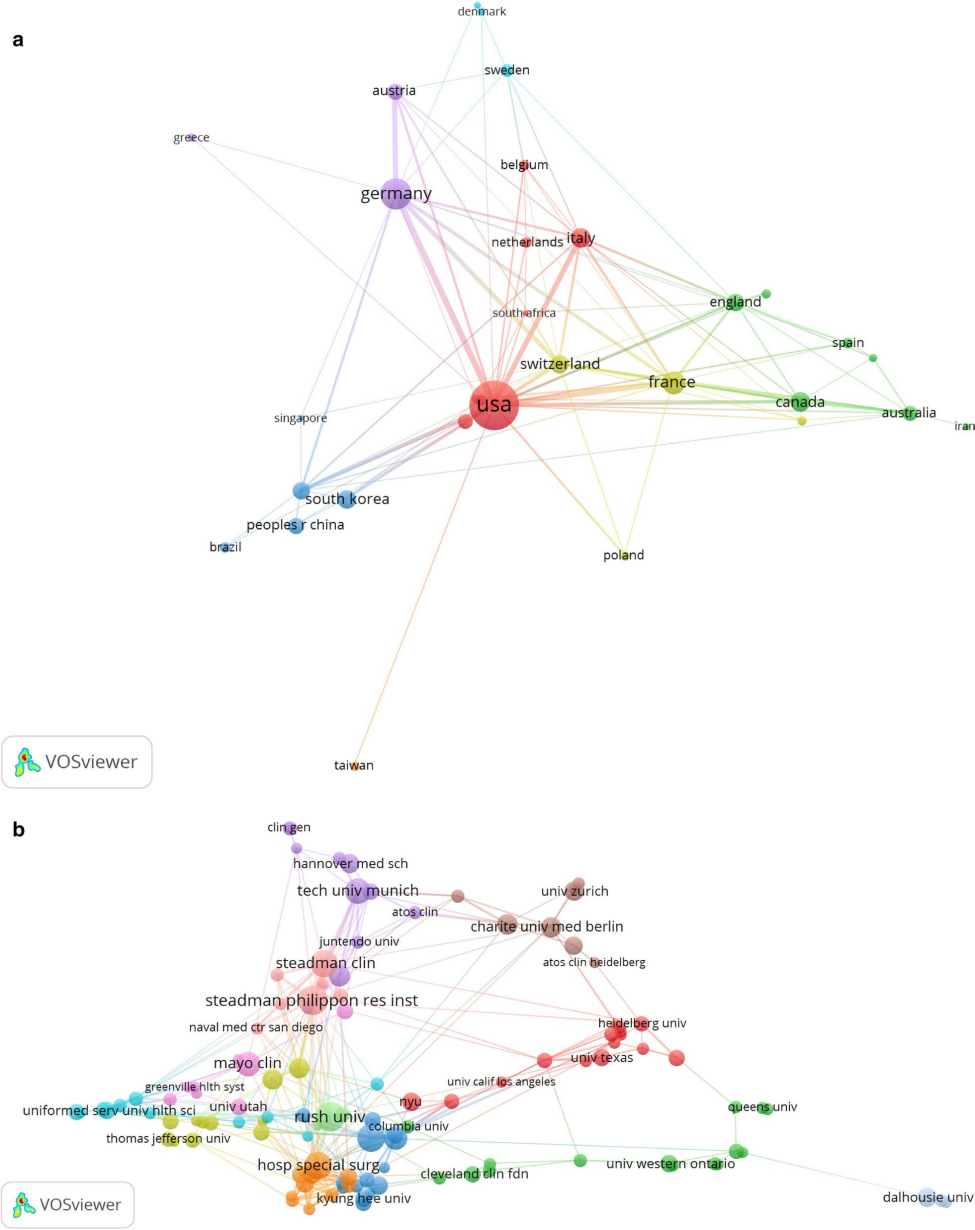

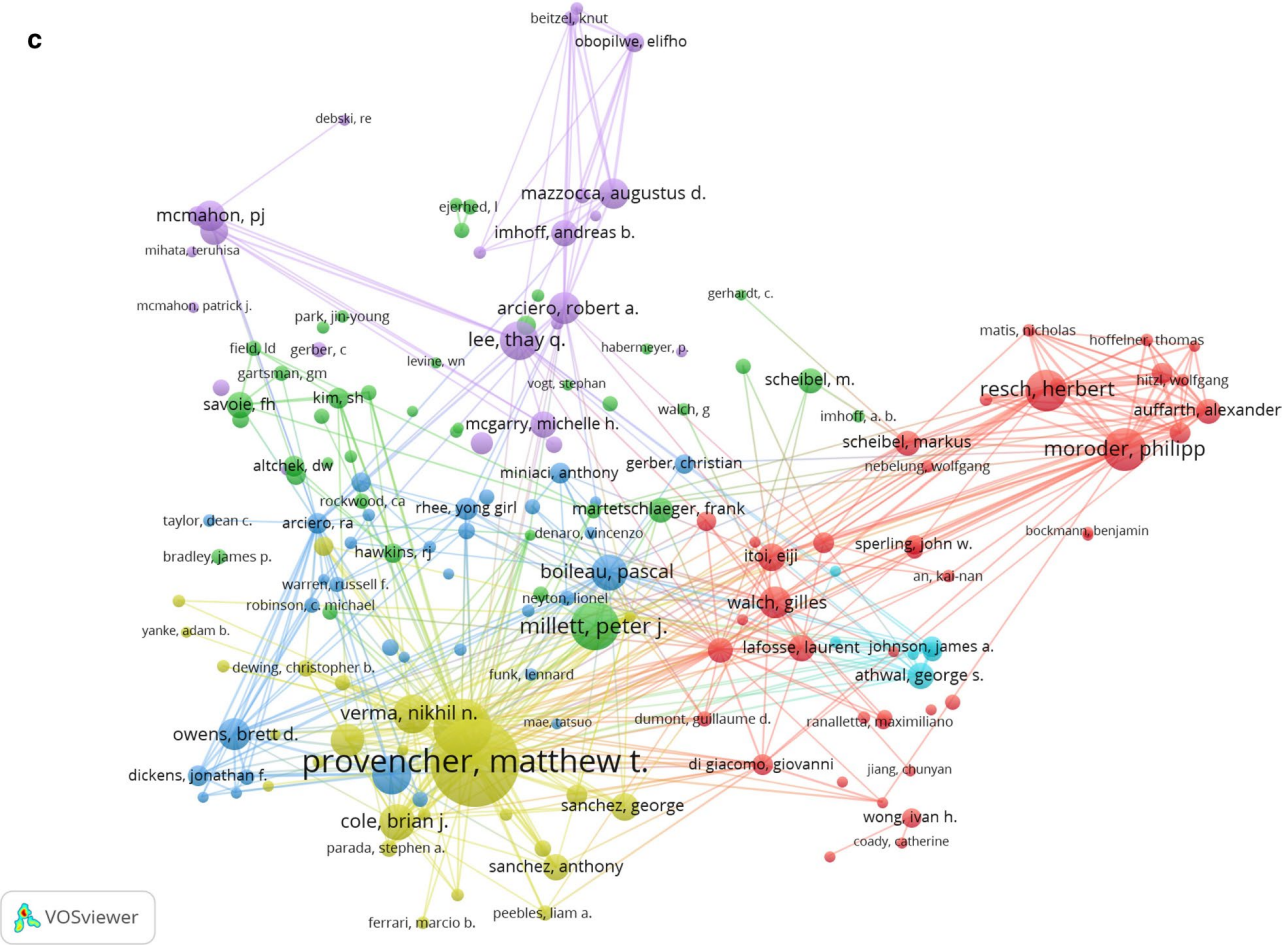
**a** The network map of countries/regions. **b** The network map of institutions. **c** The network map of authors

The statistical analysis showed that 1865 institutions from 59 countries have published articles related to the treatment of anterior shoulder instability. The United States had the most publications (44.5%). The cooperation relationships between countries are shown in Fig. 4a. Figure 4a indicates that the United States attached great importance to cooperation and had close collaborations with Germany, France, Italy, and South Korea (the size of the node indicates the influence of countries, while the thickness of the connection represents the closeness of the cooperation). The United States played an important role in the treatment of anterior shoulder instability in both the data and VOS viewer analyses. The institution with the most publications was the United States Department of Defense 65 (3.3%), followed by the University of California System 59 (3.0%) and Harvard University 58 (2.9%). The connections between these institutions are shown in Fig. 4b. The largest node was the Steadman Philippon Research Institute, which had a close collaboration with the Steadman Clinical Institute. The numbers and proportions of different countries and institutions are shown in Table 2.

**Table 2:** The top 10 countries and institutions in the anterior shoulder instability

Rank	Country/region	Count (%)	Institution	Count (%)
1	United states	875 (44.55%)	United States Department of Defense	65 (3.31%)
2	Germany	275 (14.00%)	University of California System	59 (3.00%)
3	France	137 (6.97%)	Harvard University	58 (2.95%)
4	Canada	92 (4.68%)	Rush University	58 (2.95%)
5	Italy	84 (4.27%)	Steadman Philippon Research Institute	57 (2.90%)
6	Switzerland	77 (3.92%)	Pennsylvania Commonwealth System of Higher Education Pcshe	56 (2.85%)
7	South Korea	74 (3.76%)	University of Pittsburgh	46 (2.34%)
8	Japan	69 (3.51%)	Charite Medical University of Berlin	45 (2.29%)
9	England	65 (3.31%)	Free University of Berlin	45 (2.29%)
10	Austria	51 (2.59%)	Hosp Special Surg	45 (2.29%)

### Active authors

A total of 5486 authors published 1964 articles related to the treatment of anterior shoulder instability. The top 10 authors are listed in Table 3. The author with the most publications is Provencher MT (51 articles), followed by Romeo AA (39) and Lee TQ (34). In addition, the authors with the top 10 citations are listed in Table 3. The network of authors contributing to the treatment of anterior shoulder instability is shown in Fig. 4c. The largest node was that for Provencher MT, who had the most publications and whose works laid the foundation for anterior shoulder instability research. Romeo AA was the second most highly published author.

**Table 3:** Ranking of the top 10 authors and co-cited authors in the anterior shoulder instability

Rank	Author	Frequency	Author	Citations
1	Provencher MT	51	Arcieio RA	1378
2	Romeo AA	39	Burkhart SS	1337
3	Lee TQ	34	Boileau P	1270
4	Millett PJ	28	Provencher M	1066
5	Arciero RA	26	Romeo A	1056
6	Moroder P	26	Burkhart Stephen S	956
7	Resch	26	Itoi E	853
8	Scheibel M	26	Kim SH	815
9	Walch G	24	Rockwood CA	801
10	Boileau P	23	Hovelius L	792

## Keyword co-occurrence and burst

**Fig. 5:**
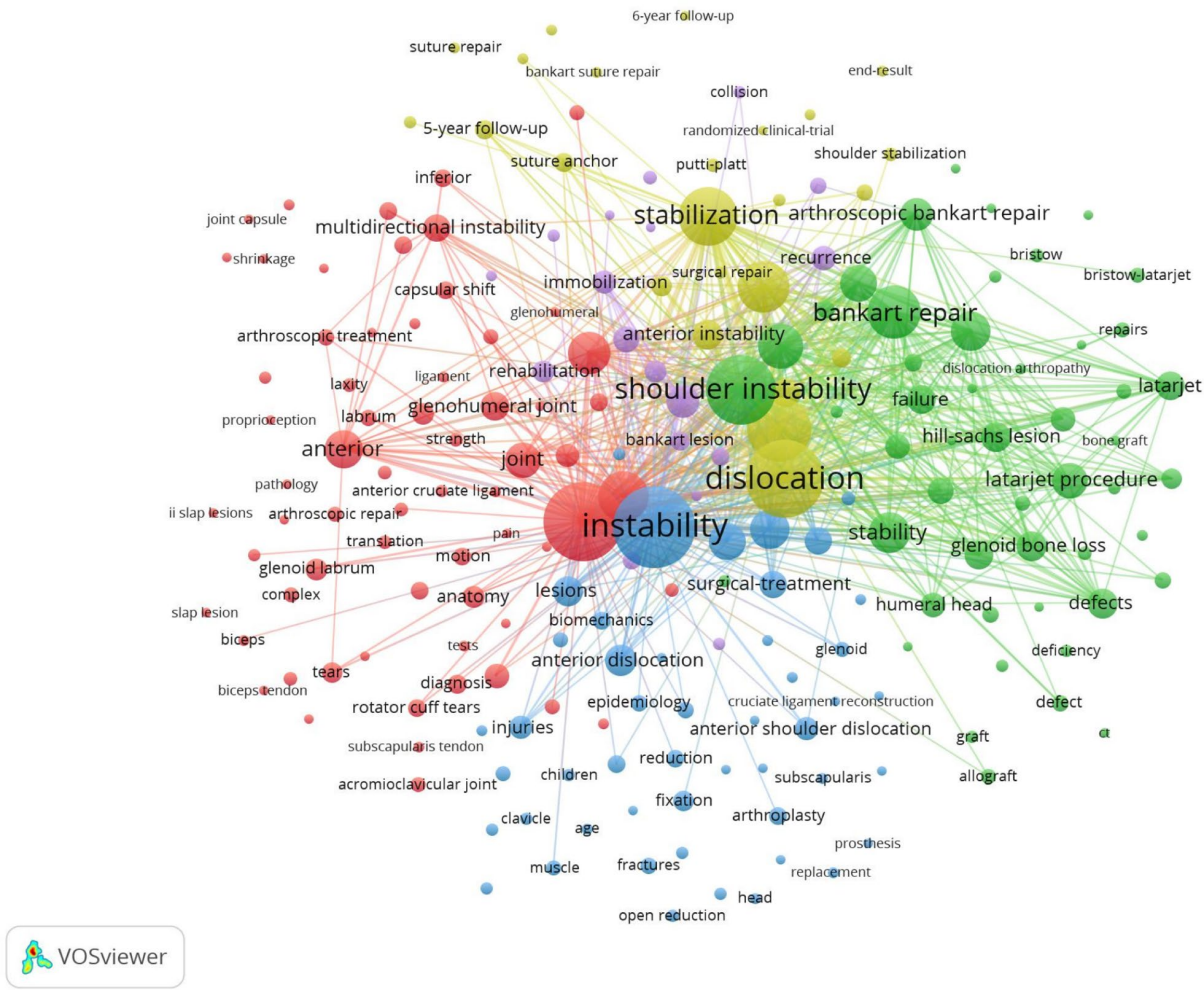
The network map of keywords

The distribution of keywords was analyzed using VOS viewer software. The minimum number of keyword occurrences was 20. The 155 keywords that met the threshold were divided into five groups. The keyword with the highest frequency was instability (545), followed by shoulder (511), and dislocation (503) (Fig. 5). As shown in Fig. 5, the largest node is instability followed by shoulder and dislocation, with an important connection between them. In addition, the keywords were identified and analyzed using Citespace’s strong citation bursts (Table 4). The keywords with strong bursts between 1984 and 2020 were “arthroscopic Bankart repair” (2014–2020), “Latarjet procedure” (2016–2020), “risk factor” (2016–2020), “recurrence” (2017–2020), and “complication” (2016–2020).

**Table 4:**
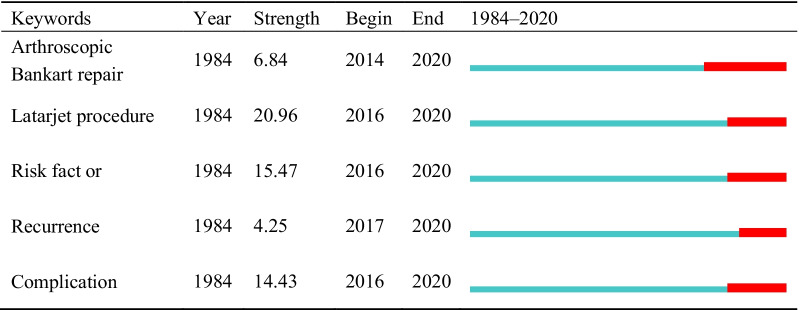
The keywords with the strongest citation bursts

## Discussion

### General information

Bibliometric analysis was used to comprehensively reveal the trends and development of publications in the field of the treatment of anterior shoulder instability, which are helpful in analyzing the current status and hotspots.

The United States showed the strongest research strength in the treatment of anterior shoulder instability, accounting for 44.5% of all publications and 80% of the top 10 journals. An important factor may be the increase in the incidence of anterior shoulder instability in the United States [3, 10]. Moreover, the United States has better scientific research power, with thousands of research institutions compared to other countries. Germany has also played an important role in promoting this field. In addition, the top 10 institutions published 534 articles from 1984 to 2020, accounting for 27.2% of all publications. The institution with the most publications was the United States Department of Defense, reflecting its influence in the treatment of anterior shoulder instability.

Among the top 10 journals, eight originated in the USA. This reflects the high level of medical care and scientific research capabilities in the United States. In addition, four journals had an IF that exceeded 4. Among them, the *American Journal of Sports Medicine* journal had the highest impact factor and published the most articles, reflecting the international influence of this journal in the field of anterior shoulder instability.

The analysis of keywords revealed those that have become and continue to be a focus in this field, including arthroscopic, Bankart repair, Latarjet procedure, risk factors, recurrence, and complications. Bankart repair and the Bristow–Latarjet procedure remain the main methods for the treatment of anterior shoulder instability. In addition, arthroscopic and complications have become the focus of research attention, with the application of the concept of minimally invasive surgery in recent years.

### Intellectual base

**Fig. 6:**
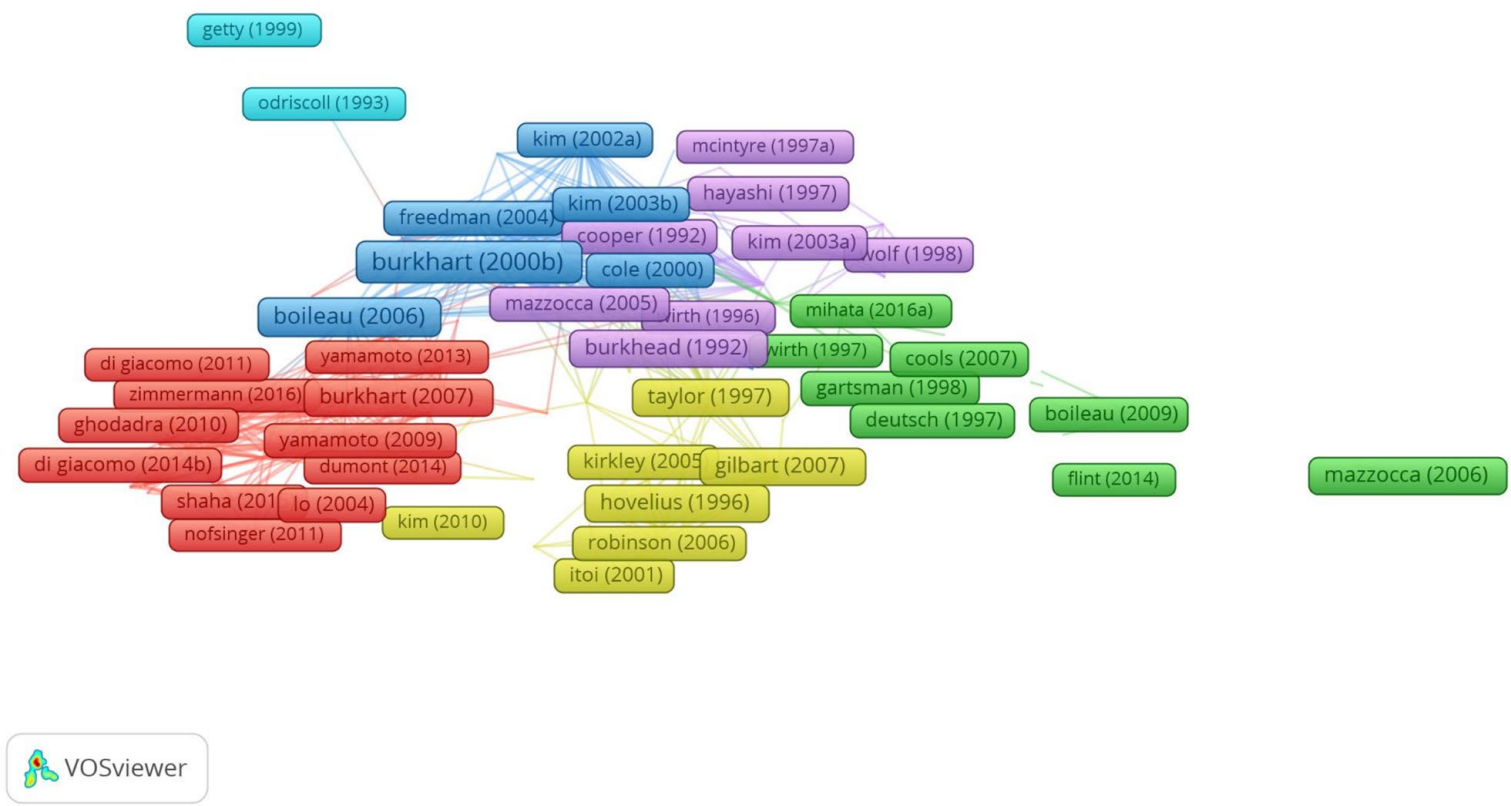
The citation network map of publications

Burkhart’s article, as the most cited paper, was located in the center of the network of citations (Fig. 6). They analyzed the effect of arthroscopic Bankart repair for anterior shoulder instability, reporting comparable results to open Bankart repairs in cases without significant bone deficits [11]. The shoulder depends on both dynamic and static stabilizers; the dynamic and static factors include soft tissue and bone, respectively [12]. The methods for the reconstruction of anterior shoulder instability are mainly divided into the Latarjet and non-Latarjet procedures, among which the Bankart repair is a representative non-Latarjet procedure. Wolf et al. [13] introduced arthroscopic Bankart repair using suture anchors for anterior shoulder instability in 1999. Glenoid bone loss is one of the most important factors related to the success of Bankart repair. Generally, glenoid bone loss above 20–25% is considered a critical factor for poor surgical outcomes after Bankart repair. However, recent studies demonstrated that labral repair alone may be inadequate to restore the stability of the shoulder in cases with 13.5–15% glenoid bone loss [14, 15]. There remains controversy regarding the optimal glenoid bone loss size, but this value has tended to decrease.

The Latarjet procedure originally described the reconstruction of shoulder stability by the dynamic sling effect of the conjoint tendon and the static effect of the transferred coracoid process in 1954 [16]. In cases with glenoid bone loss above 20–25%, the Latarjet procedure provides better results after surgery. Therefore, the Latarjet procedure has long been considered the gold standard for the treatment of anterior shoulder instability with significant glenoid bone loss. Jotyar et al. [17] compared the clinical, functional, and radiographic outcomes of open versus arthroscopic Latarjet procedures, reporting similar clinical and radiographic outcomes between them. However, the open arthroscopic Latarjet procedure had higher rates of postoperative complications such as recurrent instability, infection, musculocutaneous nerve palsy, and postoperative pain, as well as a significant learning curve.

A Hill–Sachs lesion is a bony defect of the posterolateral humeral head present in 26.7–88.1% of patients with anterior shoulder instability [18]. An insufficient understanding of Hill–Sachs lesions has led to a high rate of failure in Bankart repair [19]. Therefore, an increasing number of scholars recommend arthroscopic Bankart repair combined with Remplissage in the treatment of patients with minimal glenoid bone loss and Hill–Sachs lesions [20, 21].

Other surgical methods have also been used to treat anterior shoulder instability, such as arthroscopic subscapularis augmentation (ASA), tendon transplant, and bone grafting techniques. However, the indications for different operations require further clinical research to define.

## Research frontiers

As burst keyword analysis can reflect research frontiers to some extent, we analyzed the following burst keywords: arthroscopic, Bankart repair, Latarjet procedure, risk factors, recurrence, and complications.

Arthroscopic: With the recent development of arthroscopic techniques, shoulder arthroscopic surgery has become the gold standard for the treatment of anterior shoulder instability. The general benefits of shoulder arthroscopy include smaller incisions, lower complication rates, and faster healing compared to open surgery. Gao et al. [22] systematically analyzed the outcomes of both arthroscopic and open Bankart repairs for anterior shoulder instability, reporting that systematic studies were associated with more favorable arthroscopic outcomes. Other studies showed that the arthroscopic Latarjet procedure achieved similar results as those for open surgery in the treatment of anterior shoulder instability in both short- and mid-term follow-up [17, 23]. Therefore, arthroscopic techniques play an important role in the treatment of anterior shoulder instability. Moreover, attention is needed to improvements in arthroscopic techniques to shorten the learning curve.

Bankart repair and Latarjet procedure: The most important repairs for anterior shoulder instability were the Bankart repair and Latarjet procedures. Generally, Bankart repair is preferred when glenoid bone loss is less than 20%. However, the rate of recurrence of shoulder dislocation remains higher in patients with glenoid bone loss. In contrast, the Latarjet procedure was preferred in cases with glenoid bone loss exceeding 20–25%. Although new techniques have emerged in recent years, the Bankart repair and Latarjet procedures remain the main methods. The focus is on the selection of surgical methods for anterior shoulder instability according to glenoid bone loss.

Risk factors and recurrence: The reported rates of recurrent shoulder dislocation after the first traumatic anterior shoulder instability event were as high as 100% in adolescents [24]. When the first traumatic anterior shoulder instability event develops into recurrent shoulder instability, the financial costs, family burden, and patient pain can be substantial. Some authors argued that it is necessary to identify modifiable risk factors for recurrence of anterior shoulder dislocation following the first traumatic anterior shoulder instability event [25, 26]. Molds et al. [27] reported that age, sex, time at initial dislocation, greater tuberosity, and hyperlaxity were crucial risk factors for anterior shoulder instability and that Hill Sachs lesions, bone Bankart lesions, and nerve palsy also influenced the rate of recurrence of anterior shoulder dislocation. Therefore, risk factors and recurrence have become a research focus in recent years.

Complications: Recent technological advancements have resulted in the application of a variety of bone block procedures and soft tissue methods for the treatment of anterior shoulder instability. However, regardless of open surgery or arthroscopic procedure, the associated risks of complications remain poorly defined. Huw et al. [28] reported a tenfold higher complication rate for bone block stabilization procedures compared to soft tissue methods. Therefore, soft tissue methods such as ASA, Remplissage, and “Sling” procedures will gradually become future focus.

### Strengths and limitations

To our knowledge, this is the first bibliometric analysis of the treatment of anterior shoulder instability. In our study, we downloaded most of the articles in the field of anterior shoulder instability from the WOS and reported more objective and comprehensive results. In addition, our findings revealed the hotspots and frontiers on this topic. However, our study had some limitations. First, the data analyzed were only retrieved from the WOS, although it was representative; thus, the analysis results may differ from the actual situation. Another limitation was the restriction of articles to only English language publications, which may have led to the omission of some information. A limitation of both CiteSpace and the VOS viewer is that self-citation cannot be evaluated. Finally, since the WoSCC database only provides data from 1980 to 2020, articles before 1980 could not be searched. It is important to note that the results of our study are credible.

## Conclusions

The treatment of anterior shoulder instability has achieved great progress and shown an increasing number of publications each year. The core journal is the *American Journal of Sports Medicine*. The United States Department of Defense was the institution with the most publications. The United States is ahead of other countries in terms of the number of publications and the quality of articles. Arcieio and Burkhart are prominent researchers in this field. Bibliometric analysis of the literature on the treatment of anterior shoulder is of great significance for researchers in determining cooperative relationships, discovering research hotspots, and predicting the frontiers of treatment for the anterior shoulder.

## Data Availability

The datasets generated during and/or analyzed during the current study are available from the corresponding author on reasonable request.

## Abbreviations

WoSCC: Web of science core collection
ASA: Arthroscopic subscapularis augmentation
